# Shared and Context-Specific Mechanisms of EMT and Cellular Plasticity in Cancer and Fibrotic Diseases

**DOI:** 10.3390/ijms26199476

**Published:** 2025-09-27

**Authors:** Victor Alexandre F. Bastos, Aline Gomes de Souza, Virginia C. Silvestrini Guedes, Thúlio M. Cunha

**Affiliations:** 1 Laboratory of Experimental Biotechnology, Institute of Biotechnology, Federal University of Uberlândia, Uberlândia 38402-022, MG, Brazil; victor.bastos@ufu.br; 2 Department of Medical Imaging, Hematology, and Oncology, Ribeirao Preto Medical School, University of Sao Paulo, Ribeirao Preto 14040-900, SP, Brazil; virginiacsilvestrini@usp.br; 3 Laboratory of Nanobiotechnology, Institute of Biotechnology, Federal University of Uberlândia, Uberlândia 38402-022, MG, Brazil; thcunha@yahoo.com.br; 4 School of Medicine, Federal University of Uberlândia, Uberlândia 38402-022, MG, Brazil

**Keywords:** epithelial–mesenchymal transition, cellular plasticity, fibrosis, cancer, transcription factors, microRNAs, TGF-β signaling

## Abstract

Cellular plasticity enables cells to dynamically adapt their phenotype in response to environmental cues, a process central to development, tissue repair, and disease. Among the most studied plasticity programs is epithelial–mesenchymal transition (EMT), a transcriptionally controlled process by which epithelial cells acquire mesenchymal traits. Originally described in embryogenesis, EMT is now recognized as a key driver in both tumor progression and fibrotic remodeling. In cancer, EMT and hybrid epithelial/mesenchymal (E/M) states promote invasion, metastasis, stemness, therapy resistance, and immune evasion. In fibrotic diseases, partial EMT (pEMT) contributes to fibroblast activation and excessive extracellular matrix deposition, sustaining organ dysfunction mainly in the kidney, liver, lung, and heart. This review integrates recent findings on the molecular regulation of EMT, including signaling pathways (TGF-β, WNT, NOTCH, HIPPO), transcription factors (SNAIL, ZEB, TWIST), and regulatory layers involving microRNAs and epigenetic modifications. Moreover, we discuss the emergence of pEMT states as drivers of phenotypic plasticity, functional heterogeneity, and poor prognosis. By comparing EMT in cancer and fibrosis, we reveal shared mechanisms and disease-specific features, emphasizing the translational relevance of targeting EMT plasticity. Finally, we explore how cutting-edge technologies, such as single-cell transcriptomics and lineage tracing, are reshaping our understanding of EMT across pathological contexts.

## 1. Introduction

Cellular plasticity refers to the ability of cells to alter their phenotypic identity in response to environmental cues, even in the absence of genetic alterations. This dynamic capacity, crucial for embryonic development and tissue regeneration, is mediated by processes such as transdifferentiation, dedifferentiation, and partial phenotypic transitions [1,2]. One of the most extensively studied mechanisms of cellular plasticity is the EMT, a tightly regulated process in which epithelial cells progressively lose their apical–basal polarity and cell–cell adhesion while acquiring mesenchymal traits such as spindle-shaped morphology, increased migratory capacity, and secretion of extracellular matrix (ECM) components [3,4]. Originally described in the context of embryonic development and wound healing, EMT is now recognized as a central mechanism in a wide range of physiological and pathological conditions, including cancer and fibrosis [5,6].

In fibrogenesis, particularly in organs such as the lung, kidney, liver, and heart, it was initially believed that EMT contributed directly to the accumulation of myofibroblasts derived from epithelial cells, thereby promoting excessive ECM deposition and tissue dysfunction [5,7]. However, lineage-tracing studies have since demonstrated that epithelial cells rarely convert into myofibroblasts in vivo. Instead, they frequently adopt pEMT states characterized by a secretory, profibrotic phenotype, which, via paracrine signaling, activates resident fibroblasts, pericytes, and stromal populations, sustaining fibrosis through indirect mechanisms [8,9,10].

In cancer, EMT is associated with the acquisition of aggressive traits by tumor cells, including invasiveness, therapy resistance, metastatic potential, and immune evasion [11,12]. These conditions collectively represent some of the leading causes of global morbidity and mortality, underscoring the importance of understanding EMT across pathological contexts.

Moreover, recent studies have demonstrated that EMT does not occur as a binary switch between epithelial and mesenchymal states, but rather as a spectrum of intermediate cellular phenotypes, often referred to as hybrid or pEMT states, which endow cells with enhanced functional plasticity in both tumors and fibrotic tissues [4,5,13]. These hybrid cells coexist with other subpopulations within tumors or chronically injured tissues, contributing to phenotypic heterogeneity and complicating therapeutic responses [1,2].

Despite their distinct clinical outcomes, uncontrolled proliferation, and metastasis in cancer versus maladaptive scarring and organ failure in fibrosis, both pathologies share overlapping but context-dependent regulatory networks. At the molecular level, EMT is orchestrated by complex signaling networks including TGF-β, WNT, NOTCH, HIPPO, and inflammatory pathways, which converge on the activation of key transcription factors such as SNAIL, SLUG, TWIST, ZEB1, and ZEB2 [1,3,12]. These transcription factors repress the expression of epithelial genes such as E-cadherin, while inducing the expression of mesenchymal markers such as vimentin, N-cadherin, and α-SMA, thereby promoting both morphological and functional changes [13,14]. Integrating the fields of fibrosis and cancer through the lens of EMT-driven plasticity is not only conceptually sound but also clinically imperative. Both processes share common cellular trajectories and molecular regulators, and therapeutic strategies developed in one context may inform or inspire advances in the other [5,13]. With recent advances in single-cell technologies, spatial transcriptomics, and lineage tracing, it is now possible to precisely map and interrogate the dynamics of cellular plasticity across different tissues and disease contexts [2].

In this context, this review proposes an integrative approach to EMT and cellular plasticity, highlighting shared regulatory mechanisms and their pathological and therapeutic implications in diseases such as cancer and fibrosis.

## 2. Core Molecular Mechanisms of EMT

Beyond its phenotypic definition, EMT is sustained by a multilayered regulatory architecture that integrates extracellular signaling with transcriptional and epigenetic programs [15,16]. Central signaling pathways such as TGF-β, WNT, NOTCH, and HIPPO converge on a core set of EMT transcription factors (SNAIL, SLUG, TWIST, ZEB1, and ZEB2), which in turn repress epithelial genes while inducing mesenchymal markers [17,18]. These transcriptional changes are reinforced by non-coding RNAs and chromatin remodeling, stabilizing context-dependent cell-state transitions [19,20,21,22]. Understanding how these regulatory modules interact provides the basis to compare EMT dynamics in cancer and fibrotic diseases.

### 2.1. Extracellular Signaling Pathways

Among the key upstream regulators of EMT are the TGF-β, WNT, NOTCH, and HIPPO signaling pathways, which are highly conserved across species and frequently activated under pathological conditions. TGF-β is one of the primary inducers of EMT in the contexts of development, fibrosis, and cancer. Upon activation, it promotes the phosphorylation of SMAD2/3, which complex with SMAD4 and translocate to the nucleus, where they induce the expression of pro-EMT transcription factors such as SNAIL, ZEB1, and TWIST [5,6]. In fibrotic diseases, persistent TGF-β activity promotes myofibroblast accumulation and excessive ECM deposition, as demonstrated in idiopathic pulmonary fibrosis (IPF), chronic kidney disease, liver cirrhosis, and cardiac remodeling. In cancer, TGF-β exhibits a dual role: tumor-suppressive in early stages, but pro-tumorigenic once cells acquire oncogenic mutations. In advanced tumors, it drives EMT, invasion, stemness, angiogenesis, and immune evasion [23,24,25]. The WNT/β-catenin pathway plays a key role in maintaining mesenchymal traits and stabilizing the post-EMT phenotype. Nuclear accumulation of β-catenin leads to the transcriptional activation of mesenchymal genes and repression of epithelial genes [6,26]. In cancer, β-catenin cooperates with TCF/LEF transcription factors to induce the expression of EMT-TFs and stemness-associated genes, with strong evidence in colorectal, breast, and renal cell carcinomas. Crosstalk between WNT and TGF-β sustains hybrid EMT states, increasing heterogeneity and therapy resistance. In fibrotic tissues, WNT signaling is persistently activated during chronic injury, driving epithelial cell plasticity and fibroblast activation, particularly in IPF and renal fibrosis. Importantly, WNT inhibitors developed for oncology are now being evaluated as antifibrotic agents, underscoring shared molecular drivers [15]. NOTCH signaling, in turn, cooperates with TGF-β to promote EMT, particularly in cancer stem cells (CSC)and during metastatic progression. It directly regulates the transcription of key EMT drivers such as SNAIL and ZEB [27]. Notch is activated through ligand–receptor interactions that release the NOTCH intracellular domain, which regulates transcription of EMT and stemness genes. In fibrosis, Notch activation is strongly linked to epithelial plasticity and fibroblast expansion in renal and pulmonary injury models, while in hepatic fibrosis, it cooperates with TGF-β to sustain hepatocyte dedifferentiation. In cancer, NOTCH exerts context-dependent roles, functioning as an oncogene in breast, pancreatic, and lung tumors, but occasionally as a tumor suppressor depending on tissue and mutation status. The HIPPO pathway, through its effectors YAP and TAZ, plays a dual role in EMT regulation; it can either promote or suppress EMT depending on the tissue context and its crosstalk with other signaling pathways [12,28]. When HIPPO signaling is inactive, YAP/TAZ accumulates in the nucleus and cooperates with EMT-TFs. In fibrotic diseases, YAP/TAZ are required for fibroblast activation and persistence of myofibroblasts, as shown in lungs, kidney, and cardiac fibrosis, where mechanical stress and matrix stiffening reinforce their activity. In cancer, YAP/TAZ enhances tumor aggressiveness, metabolic reprogramming, angiogenesis, and metastatic dissemination, often cooperating with TGF-β and WNT [17,18,29,30]. These signaling pathways can be activated by stimuli such as chronic inflammation, oxidative stress, hyperglycemia, hypoxia, and interactions with an altered ECM, particularly within the tumor microenvironment and fibrotic tissues [5].

### 2.2. Master Transcription Factors

At the core of EMT regulation lies a triad of transcription factors comprising SNAIL, TWIST, and ZEB, which act synergistically to repress epithelial phenotypes and activate mesenchymal gene networks. The SNAIL family (including SNAIL1/SNAI1 and SLUG/SNAI2) promotes repression of E-cadherin (CDH1) by recruiting corepressor complexes such as HDACs, G9A, and SIN3A, leading to the disassembly of cell–cell junctions [12,31]. Beyond junctional repression, SNAIL factors are strongly implicated in tumor invasiveness and chemoresistance, particularly in breast, colorectal, and hepatocellular carcinoma [32,33,34]. In fibrotic diseases, SNAIL1 is persistently expressed in epithelial cells during pEMT. In the kidney, reactivation of SNAIL1 in tubular epithelial cells drives fibrogenesis by eliciting pEMT and paracrine activation of interstitial fibroblasts, without direct conversion into myofibroblasts, and this process can be pharmacologically mitigated [35]. In the lung, SNAIL1 contributes to pEMT in alveolar epithelial type II cells in silica-induced fibrosis models with similar upregulation and profibrotic roles observed in IPF epithelial cell populations [36,37].

ZEB1 and ZEB2 also repress CDH1 and genes involved in maintaining epithelial polarity. Additionally, they contribute to the suppression of epithelial microRNAs, such as the miR-200 family, establishing positive feedback loops that help sustain the mesenchymal state [3,6]. In carcinomas, ZEB1 drives stemness and potently mediates immune evasion. For instance, in melanoma, high ZEB1 expression suppresses CD8^+^ T-cell recruitment by repressing chemokines like CXCL10, thereby promoting tumor immune escape and resistance to PD-1 blockade [38,39]. In fibrotic contexts, ZEB1 mediates maladaptive epithelial reprogramming: in kidney, liver, and lung injury models, epithelial cells expressing ZEB1 develop a profibrotic secretory phenotype that activates mesenchymal cells and exacerbates ECM deposition [40,41]. The ZEB/miR-200 axis is especially relevant to maintaining intermediate EMT states that underline phenotypic plasticity. This axis is implicated in metastatic relapses in cancer and progression of IPF, highlighting its role in diseases marked by plasticity-driven pathology [42].

Finally, TWIST1 and TWIST2, members of the basic helix–loop–helix (bHLH) transcription factor family, act primarily during the later stages of EMT, where they facilitate cell migration, invasion, and ECM remodeling [11,12,43]. TWIST1 is frequently upregulated in aggressive tumors, including neuroblastoma, gastric, breast, prostate, and hepatocellular carcinoma, where its expression correlates with poor prognosis and therapy resistance [44,45]. Mechanistically, TWIST1 promotes stemness programs, epithelial plasticity, and metastatic dissemination. TWIST2, while less studied, has been shown to play context-dependent roles, functioning as an EMT enhancer in colorectal and pancreatic cancers [45].

In fibrotic disease, TWIST1 contributes to maladaptive tissue remodeling by promoting fibroblast activation, collagen deposition, and persistence of myofibroblasts [46,47]. In models of cardiac fibrosis, TWIST1 is induced downstream of TGF-β and angiotensin II signaling, directly regulating fibroblast contractility and ECM production [48,49]. In pulmonary fibrosis, TWIST1 expression in alveolar epithelial cells and fibroblasts sustains profibrotic gene programs and facilitates epithelial–mesenchymal plasticity (EMP), bridging chronic inflammation and progressive scarring [50,51].

These transcription factors operate in concert with their dosage, timing, and co-expression tuned by upstream pathways and tissue context. In many cases, intermediate EMT states involve partial or transient expression of these factors, resulting in hybrid phenotypes with high plasticity [11].

### 2.3. MicroRNAs and Epigenetic Regulation

EMT is also deeply modulated by microRNAs and epigenetic modifications, which can either reinforce or inhibit cellular transition [22,52]. The miR-200 family plays a pivotal role in maintaining the epithelial phenotype by repressing ZEB1 and ZEB2. During EMT, ZEB factors suppress *miR-200* expression, establishing a mutual double-negative feedback loop that governs the phenotypic switch and maintains bistability in cell fate decisions [12]. This regulatory circuit has been extensively validated in breast and colorectal cancers, where loss of miR-200 correlates with metastasis and poor prognosis [53,54]. In fibrotic disorders, reduced miR-200 expression has been documented in kidney and lung tissues, contributing to persistent fibroblast activation and ECM accumulation [55,56]. Other microRNAs, such as miR-34 (a known transcriptional target of SNAIL) and miR-203, also regulate EMT-TFs at the post-transcriptional level. These miRNAs form intricate regulatory networks that modulate EMT dynamics and represent promising therapeutic targets for both cancer and fibrotic diseases [6]. For example, miR-34 suppresses SNAIL-driven EMT in tumors, acting as a tumor suppressor, while in pulmonary fibrosis, its downregulation enhances epithelial plasticity [57,58]. Similarly, miR-203 loss is associated with the aggressiveness of hepatocellular carcinoma and with TGF-β–driven renal fibrosis [59].

At the epigenetic level, mechanisms such as DNA methylation, histone modifications, and global chromatin remodeling contribute to the repression of epithelial genes and activation of mesenchymal programs. These changes can be directly induced by TGF-β signaling or indirectly through the recruitment of chromatin modifiers by transcription factors like SNAIL and ZEB [2,12,13]. EZH2-mediated H3K27 trimethylation and HDAC-driven chromatin compaction are recurrent in tumors with high EMT activity, while in fibrosis, these mechanisms establish a “fibrotic memory” in fibroblasts, perpetuating maladaptive activation even after the injurious stimulus has ceased [52,60]. Epigenetic inhibitors such as DNMT and HDAC inhibitors have therefore emerged as candidate therapies for both cancer and fibrotic diseases [61,62].

Recent studies in pancreatic and breast cancers have shown that EMT is closely associated with increased genomic instability, particularly in cells exhibiting high mesenchymal plasticity. This instability may accelerate tumor evolution, leading to the emergence of more aggressive subclonal populations [4,12]. Although less explored in fibrosis, accumulating evidence suggests that epigenetic instability in epithelial cells undergoing EMT can predispose tissues to maladaptive scarring and progressive organ dysfunction, bridging cancer biology and chronic fibrotic disease [52,63].

Taken together, extracellular signaling pathways, EMT-inducing transcription factors, and microRNA/epigenetic networks form an interconnected regulatory architecture that enables epithelial cells to undergo profound phenotypic reprogramming. While these multilayered mechanisms are shared across pathological contexts, their downstream consequences diverge. Recognizing both the shared frameworks and the context-specific outputs of EMT regulation is essential, not only for a deeper understanding of disease biology but also for identifying actionable biomarkers and therapeutic targets in cancer and fibrosis.

**Figure 1:**
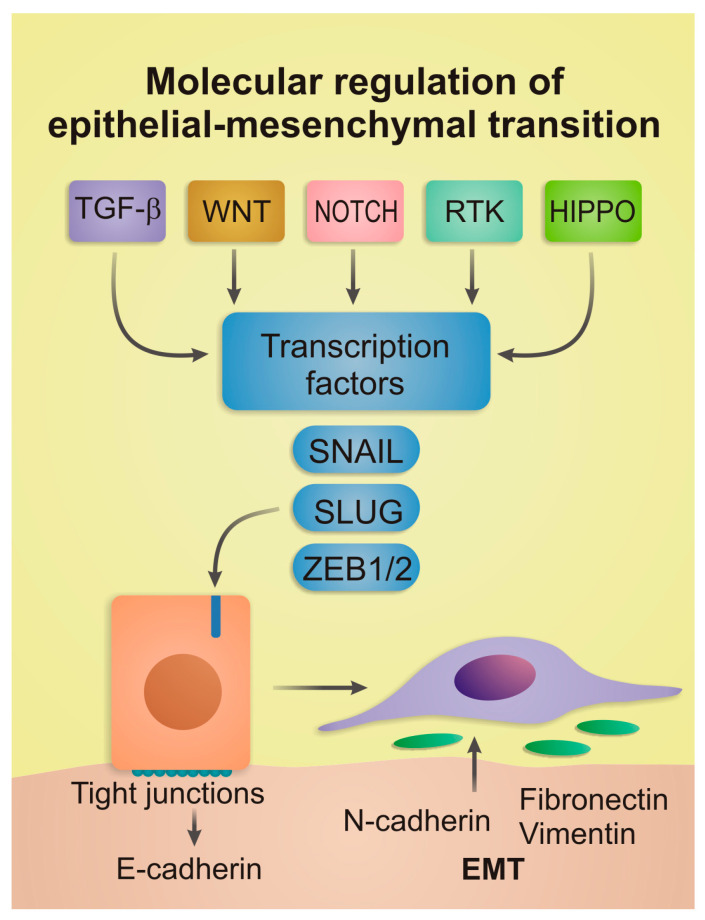
Molecular regulations of EMT. Core upstream pathways (TGF-β, WNT, NOTCH, HIPPO), downstream EMT-transcription factors (SNAIL/SLUG, ZEB, TWIST), and modulators such as non-coding RNAs and epigenetic regulators are shown as integrated drivers of EMT and pEMT states.

A consolidated schematic of EMT regulation is shown in Figure 1. The diagram integrates upstream signaling (TGF-β, WNT, NOTCH, RTK, HIPPO/YAP–TAZ), core transcription factors (SNAIL/SLUG, ZEB1/2, TWIST1/2), and canonical E/M outputs.

## 3. EMT and Cancer Plasticity

EMP is a pervasive feature of human tumors and rarely operates as a binary switch. Across cancer types, tumor cells populate a continuum of epithelial, hybrid E/M, and mesenchymal states that are tuned by microenvironmental cues and evolve along distinct EMT trajectories with prognostic and therapeutic implications [64]. Hybrid E/M states are common in patient tumors and associate with increased aneuploidy, immune–stromal interactions, and invasive behavior, while spatial profiling shows enrichment of EMT programs at leading edges across cancers [65,66]. Together, these observations position EMP as a systems-level driver of heterogeneity, dissemination, and treatment failure [64].

### 3.1. Partial and Hybrid EMT Phenotypes

In cancer, EMP rarely behaves as an all-or-none switch, but a spectrum of epithelial–mesenchymal phenotypes, many of which never progress to a fully mesenchymal profile [67,68]. These hybrid phenotypes have been recurrently identified across diverse tumors such as breast, lung, colorectal, and renal carcinomas, and are especially enriched at invasive fronts and among circulating tumor cell (CTC) clusters, facilitating metastatic dissemination [11,66].

In breast cancer, hybrid E/M states are prominent in triple-negative breast cancers (TNBC) compared with luminal tumors and map to invasive fronts on spatial and single-cell profiling. These hybrid cells co-express epithelial junctional and cytokeratin programs with mesenchymal motility and ECM modules, correlating with therapy tolerance and stem-like behaviors. Systematic surveys of EMP further show that intermediate phenotypes are common and functionally competent, rather than mere transitory states. Together, these data support a model in which pEMT expands phenotypic options that fuel invasion and recurrence in aggressive breast cancer [11,69].

In lung adenocarcinoma, spatial transcriptomics delineates EMT gradients that peak at tumor–stroma interfaces and perivascular niches, where TGF-β, hypoxia, and matrix cues shape the balance between epithelial and mesenchymal programs, favoring hybrid E/M states. Pan-cancer frameworks that quantify EMT from transcriptomes consistently detect multiple intermediate states in individual tumors, linking higher EMT scores to invasive growth and immune evasion. Spatial studies of leading-edge epithelium corroborate this gradient architecture and outcome, with transitional programs concentrated at the edge rather than the core. Functionally, these hybrid states are implicated in collective invasion and in the seeding of therapy-tolerant cell populations [65,66].

In another example, colorectal cancer exhibits epithelial intrinsic subtypes (iCMS2/iCMS3) that refine the CMS taxonomy; iCMS3 tumors show worse outcomes and greater inflammatory/stem-like features [70]. Spatial multi-omic “cartography” identifies conserved differences between tumor core and leading edge, with edge regions enriched for invasion and EMT-related programs and ligand–receptor circuits that promote local spread. Although EMT-associated CMS4 signals can partly reflect stromal contributions, single-cell and spatial datasets collectively support the presence of transitional epithelial programs at invasive fronts that couple plasticity with microenvironmental cues [71,72].

Taken together, understanding the spatial and phenotypic heterogeneity of hybrid E/M states is essential for interpreting tumor behavior and therapeutic response. However, pEMT also affects stemness circuits, embedding self-renewal and dormancy within epithelial compartments and thereby shaping CSC populations, while adding another regulatory layer.

### 3.2. EMT and Cancer Stem Cells

The CSC model posits that tumors contain a subset of cells with self-renewal capacity and the ability to regenerate heterogeneous progeny. Converging evidence indicates that EMT programs, especially in partial/hybrid forms, interlock with stemness, enabling carcinoma cells to acquire CSC-like traits, including tumor initiation, stress tolerance, immune evasion, and multidrug resistance. In patient cohorts and experimental models, higher EMT scores track with CSC markers and functional readouts of stemness [73,74,75,76].

Within this framework, EMT serves as an epigenetic reprogramming mechanism, enabling differentiated tumor cells to acquire CSC-like properties, including enhanced tumor-initiating capacity, resistance to multiple therapies, and the ability to survive under hostile conditions [75,77]. These effects are most pronounced in intermediate EMT states, which preserve epithelial adhesion while activating mesenchymal survival and ECM programs, an organization that favors self-renewal, dormancy, and therapy tolerance [73,78].

CSCs have been identified in numerous human malignancies, and experimental induction of EMT in carcinoma cells has been shown to increase their tumorigenic potential [75,77].

In breast cancer, intermediate EMT clones co-express epithelial and mesenchymal modules and display enhanced tumorigenicity, metastasis, and recurrence in vivo, establishing a direct functional link between pEMT and stemness-driven progression [78,79].

In pancreatic ductal adenocarcinoma, EMT programs intertwined with AXL signaling sustain self-renewal and long-term tumor maintenance, contributing to aggressive biology and drug resistance [80].

These observations underscore the interdependence between EMT-mediated plasticity and the CSC state, highlighting a major therapeutic challenge: the need to design strategies capable of eradicating these plastic, therapy-resistant cell populations to achieve durable clinical responses [81,82,83].

### 3.3. Functional Consequences: Invasion, Metastasis, and Therapy Resistance

The transition from a localized primary tumor to disseminated disease is a multistep process involving complex interactions between cancer cells and their surrounding microenvironment. This process, often referred to as the metastatic cascade, begins with the local invasion of tumor cells into the surrounding stroma, followed by intravasation into the circulatory or lymphatic systems, survival during transit, extravasation into distant tissues, and eventual colonization to form secondary tumors [84,85].

A hallmark event enabling invasion is the loss of epithelial characteristics and acquisition of mesenchymal traits through EMT or hybrid E/M states. These phenotypic changes confer increased motility, resistance to detachment-induced apoptosis, and the ability to degrade and remodel the ECM via secretion of matrix metalloproteinases (MMPs) and other proteolytic enzymes [67,86]. While EMT can be induced by multiple signaling pathways, cancer cells can also exploit pEMT states that preserve certain epithelial features, enhancing collective migration and cooperative invasion [86,87].

In parallel, cancer-associated fibroblasts (CAFs), immune cells, and endothelial cells within the tumor microenvironment actively support invasion by releasing growth factors, cytokines, and ECM components that facilitate migration and protect tumor cells from immune clearance [88,89]. Integrin-mediated adhesion dynamics, cytoskeletal remodeling, and activation of Rho GTPases further enable cancer cells to navigate through the dense stromal architecture [84,86].

Metastasis is not solely dependent on cellular motility; the colonization phase at distant sites is often the rate-limiting step. Metastatic cells must adapt to new microenvironments, evade immune surveillance, and, in some cases, revert to an epithelial phenotype through mesenchymal-epithelial transition (MET) to establish macroscopic secondary tumors [90,91]. In addition, a subset of disseminated cells exhibits CSC-like properties, allowing for long-term dormancy and later reactivation, which underlies metastatic relapse years after primary tumor removal [75,77,90,91].

Beyond EMT, tumor cells switch among collective, mesenchymal, and amoeboid invasion modes, often interconverting in response to matrix architecture and stress [84,85]. Partial EMT particularly favors collective migration by retaining some cell–cell adhesion while boosting motility and matrix remodeling via MMPs and cytoskeletal control through Rho GTPases [87,92]. Effective metastasis requires a receptive microenvironment at the secondary site, the pre-metastatic niche (PMN). Through cytokines, growth factors, and extracellular vesicles (EVs), primary tumors educate resident stromal and immune compartments and prime ECM composition; EV integrin signatures help route tumor material to specific organs. Targetable axes include VEGF-dependent angiogenesis, HGF/MET, and EV–integrin binding [93,94].

The colonization step is often rate-limiting. Disseminated tumor cells (DTCs) can persist as dormant cells, quiescent, immune-controlled, or angiogenic-restricted for years, then reactivate upon microenvironmental cues, mechanical remodeling, inflammation, or niche-derived signals, forming macroscopic metastases. Within the metastatic ecosystem, CAFs diversify into functional subtypes, myofibroblastic (myCAFs), inflammatory (iCAFs), and matrix-remodeling (mCAFs), that remodel ECM, exclude immune effectors, alter drug penetration, and actively guide invasion routes [88,95]. Spatial single-cell studies now map CAF neighborhoods linked to progression and therapeutic response [95].

Therapeutically, the most promising strategies target plasticity and cooperation rather than a single pathway: (i) dampening EMT/pEMT signaling nodes, (ii) disrupting PMN formation and EV-mediated organotropism, (iii) CAF modulation to normalize/stiffen-tune ECM and improve immune/drug access, and (iv) blocking integrin–FAK and downstream motility circuits, ideally as rational combinations timed to vulnerable metastatic stages [84,89,93,96].

## 4. EMT and Plasticity in Fibrotic Diseases

Fibrotic diseases constitute a diverse group of chronic conditions characterized by progressive and excessive ECM deposition that disrupts tissue architecture and impairs organ function. Fibrosis is not organ-restricted and often represents the final common pathway of many chronic injuries [97,98]. As of 2019, fibrotic diseases accounted for approximately 17% of global deaths, rising to nearly 34% when neoplasms are included, underscoring their population-level impact [99]. Among the most prevalent fibrotic conditions, chronic kidney disease (CKD) affecting around 10–14% of the global population [100], chronic liver diseases and alcohol or virus-related cirrhosis, responsible for roughly 1.47 million deaths in 2019 [101], within cardiovascular disease, the leading global cause of death (32% of all deaths in 2022), cardiac fibrosis represents a common downstream pathway that drives ventricular dysfunction and heart-failure progression [102], and pulmonary fibrosis, especially IPF, a less prevalent, but highly lethal condition, affecting an estimated 1 to 13 per 100,000 individuals worldwide, with median survival below five years [103,104].

Across organs, EMT programs, often in partial and context-dependent forms, reconfigure injured epithelia and amplify epithelial–mesenchymal crosstalk that activates and sustains fibroblasts and myofibroblasts, driving matrix accumulation and scar progression. In line with this view, the circuitry that controls EMT, with TGF-β at its core together with inflammatory and matrix-derived cues, overlaps broadly with key fibrogenic processes, positioning EMT both as a readout of maladaptive repair and as a tractable lever across fibrotic diseases [23,105].

### 4.1. EMT and FMT: Concepts and Interactions

As previously discussed, EMT plays a pivotal role in the initiation and progression of fibrotic diseases. In affected tissues, activation of this program is often incomplete, a phenomenon referred to as pEMT. This partial nature was first suggested by observations that the loss of E-cadherin expression, a classical hallmark of epithelial dedifferentiation, typically occurs only partially. Cells exhibiting pEMT are markedly enriched in fibrotic lesions across the lung, kidney, and liver when compared with non-fibrotic tissue [7,68]. Such incomplete activation of the EMT program results in considerable phenotypic heterogeneity among epithelial-derived cells within the same lesion, enhancing their plasticity and capacity to adapt to diverse microenvironmental cues [7,9,68]. These intermediate states can confer distinct functional capacities depending on the biological context. In cancer, hybrid E/M cells often display increased invasiveness and higher metastatic potential [67,87]. Conversely, in fibrotic diseases, cells may adopt a pEMT phenotype without acquiring the invasive properties typical of metastatic cancer cells, thereby contributing primarily to tissue remodeling rather than dissemination [7,9,68].

The existence of such phenotypic gradations, combined with the dynamic and reversible nature of EMT, adds substantial complexity to its analysis and classification. Experimental models, in both physiological and pathological contexts, have revealed that EMT can progress through multiple trajectories, each associated with specific gene expression programs, cellular activities, and biological outcomes that extend beyond migration and invasion [67,92]. In fibrotic diseases, the EMT program is not only partially activated but also often reversibly engaged, allowing cells to revert via MET to epithelial states during disease progression or resolution. In many fibrotic conditions, cells at sites of active tissue remodeling frequently display signs of EMT activation, including reduced E-cadherin expression, whereas adjacent cells retain epithelial traits and maintain extensive cell–cell adhesions [7,9,68].

This coexistence of distinct phenotypic states, along with evidence of dynamic interconversions between them, supports the concept that epithelial-derived cells in fibrosis can flexibly activate EMT and MET programs in response to environmental and signaling cues. Such plasticity underlies both the persistence of fibrotic processes and their potential reversibility, offering important therapeutic implications [67,87]. In the kidney, lineage-tracing studies have demonstrated that tubular epithelial cells rarely become myofibroblasts directly; instead, they engage in pEMT and activate resident fibroblasts/pericytes (including Gli1^+^ stromal cells) via paracrine signaling. Key signaling axes in this process include the TGF β/SMAD, WNT/β catenin, NF-κB/BRD4, and the mechanosensitive HIPPO pathway, which integrates matrix stiffness and cellular stress into transcriptional reprogramming [106,107,108,109].

In pulmonary fibrosis, especially IPF, injured alveolar type II cells adopt a hybrid E/M phenotype, maintaining certain epithelial markers while expressing mesenchymal genes and secreting profibrotic mediators [105,110]. Single-cell RNA-seq studies have identified these mesenchymal-like epithelial populations, which interact with fibroblasts and respond to matrix stiffness via YAP/TAZ signaling, establishing a profibrotic feedback loop [17]. In addition, the HIPPO/YAP pathway has been implicated in the progression of IPF, modulating the balance between epithelial regeneration and fibrotic activation [111].

In the heart, cardiac fibrosis commonly follows myocardial infarction or chronic stress. Myofibroblasts derive primarily from resident fibroblasts through fibroblast-myofibroblast transition (FMT); however, endothelial-to-mesenchymal transition (EndoMT) and activation of epicardial cells or MSC-like populations also contribute [112,113,114]. Cardiomyocytes and endothelial cells may undergo pEMT/EndoMT in response to injury and chronic inflammation, promoting maladaptive remodeling and fibrosis [114].

Though most mechanistic insights come from kidney, lung, and heart fibrosis, similar plasticity mechanisms have been described in other organs. In chronic liver injury, hepatocytes and cholangiocytes activate pEMT programs that stimulate hepatic stellate cells activation through TGF-β, Hedgehog, and Notch signaling, intensifying collagen production and septal scarring [115,116]. In the skin, keratinocytes in chronic wounds and scleroderma display EMT-like plasticity linked to dermal remodeling [105]. Similarly, in pancreatitis, ductal cells express EMT-like signatures contributing to pancreatic fibrosis [117].

Together, the evidence across organs highlights EMT as a partial, plastic program that amplifies fibrosis indirectly, while FMT sustains ECM deposition. Their interplay is reinforced by chronic inflammation and the activity of mesenchymal stem cells (MSCs). Although MSCs initially support tissue repair, under sustained inflammatory conditions, they can differentiate into myofibroblasts or promote their activation through persistent profibrotic signaling [118].

### 4.2. Inflammation–MSC Axis in Fibrotic Plasticity

Chronic inflammation is a primary amplifier of fibrotic plasticity. Immune proinflammatory cues such as TGF-β, Activin A, IL-1/IL-6, type-2 and type-17 cytokines, polarize and educate tissue macrophages and other leukocytes, which in turn instruct stromal cells to adopt profibrotic states while reinforcing epithelial programs such as pEMT. This bidirectional immune–stromal dialog sustains myofibroblast activation and stabilizes scar architecture across organs [23,119].

Interestingly, the duality of MSCs sits at the heart of this process. In acute injury, MSCs can be reparative and immunomodulatory; under sustained inflammatory stress, however, resident MSC-like populations differentiate into myofibroblasts or secrete factors (TGF-β, CTGF, PDGFs) that maintain myofibroblast activity, thereby hard-wiring epithelial–stromal loops. This behavior has been documented across lungs, kidney, liver, and heart fibroses [118,120].

Furthermore, paracrine trafficking via EVs enhances inflammation to mesenchymal activation. MSC-derived EVs (MSC-EVs) deliver miRNAs and other cargo that can reprogram epithelial and stromal states, attenuating or, in inflammatory milieus, sustaining profibrotic signaling and matrix production; therapeutically engineered MSC-EVs are being explored to use this axis as a therapeutic approach [121,122].

These inflammatory and MSC-centered circuits interface tightly with epithelial plasticity. pEMT in injured epithelia enhances cytokine/chemokine and EV output, which recruits and educates MSC-like and fibroblast populations; stiffened ECM and mechanosensitive pathways then stabilize these states, closing the loop that links immune tone, mesenchymal activation, and epithelial programs in a context-dependent manner [37,123].

Organ contexts echo this shared logic. In pulmonary fibrosis, monocyte-derived macrophages and transitional AT2 populations co-operate to maintain fibroblast activation, with inflammatory priming of stromal/MSC compartments aligning with progressive scarring. In cardiac remodeling, inflammatory macrophage subsets orchestrate fibroblast phenotypes and ECM deposition after ischemia or pressure overload, while MSC-like stromal cells contribute to the persistence of myofibroblasts [119,124].

By creating redundant, mutually reinforcing routes to maintain mesenchymal activation and epithelial plasticity, the inflammation–MSC axis helps understand why fibrotic tissues remain elusive to therapy.

### 4.3. Therapy Resistance and Plasticity

In fibrotic disorders, EMT-driven plasticity contributes to intrinsic and acquired resistance to therapy. Stabilized pEMT states promote mesenchymal survival programs, including reduced apoptosis, altered metabolism, and persistent ECM production, which blunt responses to antifibrotic agents [7,125]. These states can persist even when upstream TGF-β cues are attenuated, reflecting redundancy via Wnt/β-catenin, Notch, PI3K/AKT, and other pathways [23].

Resistance is further reinforced by microenvironmental remodeling. Progressive ECM stiffening and altered integrin signaling via FAK and Rho-family GTPases create feed-forward loops that maintain myofibroblast activation and dampen drug efficacy [7,126,127]. Upstream mechanical cues (stiffness, strain) drive nuclear YAP/TAZ and reprogram cell state and transcription, locking fibrotic phenotypes despite pharmacologic inhibition of single pathways [128,129,130]. Cellular heterogeneity also undermines treatment. Fibrotic tissues harbor diverse fibroblast/myofibroblast subsets with distinct signaling dependencies and drug sensitivities, analogous to CAF states in cancer [131,132]. Single-cell atlases across organs reveal inflammatory, matrix-producing, and contractile fibroblast neighborhoods that can compensate for one another under therapy, sustaining disease [133,134,135].

Clinically, approved agents for pulmonary fibrosis (pirfenidone, nintedanib) slow functional decline but do not halt or reverse disease, making persistence or relapse common, consistent with tolerance and tissue plasticity [136,137].

In renal fibrosis, precision approaches highlight variable responses and the need for patient stratification and combinations that target plasticity and mechanics [107]. Recent studies indicate that single-cell and spatial profiling of human chronic kidney disease resolves epithelial pEMT programs and distinct fibroblast and pericyte states, enabling molecular stratification of biopsies [138,139]. They also show that blockade of TGF-β alone is insufficient, whereas co-inhibition of mechanosignaling through integrin–FAK and YAP–TAZ pathways was more effectively in reducing myofibroblast persistence and ECM deposition [107,140,141]. In parallel, epigenetic modulation with BET and BRD4 inhibitors dampens transcriptional memory of the fibrotic state and improves antifibrotic responses [108,142]. Finally, interventions directed at alpha-v integrins or at FAK can slow the transition from injury to fibrosis, supporting rational combination regimens guided by tissue-level molecular stratifiers rather than uniform therapy for all patients [143,144].

At a molecular level, epigenetic regulators and non-coding RNAs integrate TGF-β and mechanical cues to sustain resistant states. In lung fibrosis, the lncRNA DNM3OS, a reservoir of “fibromiRs”, and related ncRNAs are critical downstream effectors; interfering with this axis mitigates myofibroblast programs and may re-sensitize tissue to therapy [25,145,146].

Overcoming these resistance mechanisms will likely require multi-target regimens that curb EMT and pEMT plasticity, normalize ECM mechanics and integrin signaling, account for fibroblast subset heterogeneity, and layer epigenetic or non-coding RNA interventions, strategically timed to coincide with windows of heightened lesion plasticity [7,25,126,132,146].

## 5. Shared and Distinct Features of EMT in Cancer and Fibrosis

Although EMT operates through core molecular mechanisms across diverse biological contexts, its functional roles and pathophysiological outcomes diverge significantly between fibrotic diseases and cancer. In fibrosis, context-dependent, often pEMT, within injured epithelia primarily amplifies stromal activation and matrix remodeling, whereas in carcinomas, pEMT underpins invasion, therapy tolerance, and metastatic seeding [105,147]. Below, we summarize the key convergences and distinctions of EMT in these two scenarios.

**Figure 2:**
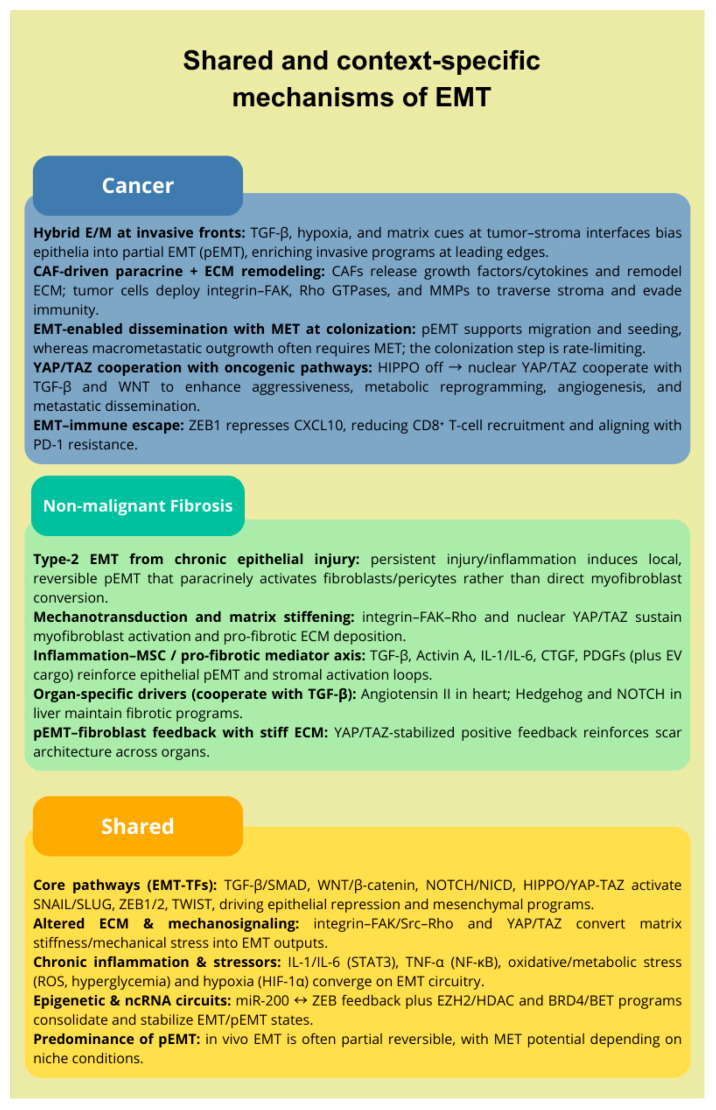
Shared and context-specific mechanisms of EMT in malignant neoplasms and non-malignant fibrosis. The figure highlights cancer-specific, fibrosis-specific, and common disease traits triggering EMT/pEMT, illustrating both distinct and overlapping mechanisms across various contexts.

Common and disease-specific traits that trigger and shape EMT in cancer versus fibrosis are synthesized in Figure 2.

### 5.1. Structural and Molecular Similarities

In both fibrosis and cancer, EMT features the repression of epithelial genes, such as CDH1 (E-cadherin), together with the induction of mesenchymal markers including VIM (vimentin), FN1 (fibronectin), and ACTA2 (α-SMA) [3,5]. These shifts are driven by conserved signaling networks, notably TGF-β, WNT/β-catenin, NOTCH, and HIPPO (YAP–TAZ), that converge on core EMT transcription factors (SNAIL, ZEB1/2, TWIST) to reprogram the transcriptome and remodel chromatin [6,12]. EMT is also capable of establishing adaptive plasticity, frequently through intermediate and reversible states that support survival in hostile microenvironments [1,2,13]. In both settings, EMT-programmed cells also remodel the secretome and deposit ECM (for example, collagens and fibronectin), contributing to structural changes and niche conditioning [5]. Moreover, epigenetic regulators and non-coding RNAs integrate these signals to stabilize EMT-linked states across contexts, reinforcing either stromal activation in fibrosis or metastatic plasticity in cancer [12,13].

### 5.2. Functional Role of EMT in Fibrosis

According to the current EMT classification, type 2 EMT refers to fibrosis-related processes, while type 3 EMT describes carcinoma-associated EMT. In fibrosis, type 2 EMT functions primarily as a maladaptive wound-healing mechanism. It is typically activated in response to chronic injury, persistent inflammation, or metabolic stress, driving epithelial cells into pEMT states that alter junctions and transcriptional programs while remaining locally confined to sites of damage [37,105]. Rather than undergoing widespread conversion into myofibroblasts, injured epithelia in pEMT reprogram their secretome and EVs cargo to activate resident fibroblasts and pericytes via paracrine signaling, thereby amplifying FMT, ECM deposition, and progressive organ dysfunction [139,148,149]. These EMT-linked epithelial states are typically local rather than migratory and can be reversible. However, the potential for EMT-associated fibrosis to regress is organ- and time-dependent. In the liver, removal of the etiologic driver is followed by histologic fibrosis regression in many patients [150]. In the lung, meaningful regression is uncommon, and current antifibrotics are capable of only slowing functional decline with limited reversal of established scar [104]. In myeloproliferative neoplasms, bone-marrow osteomyelofibrosis can also regress when the malignant clone is eradicated by allogeneic hematopoietic cell transplantation, with sequential biopsies showing downgrading of reticulin, collagen, and osteosclerosis [151]. Pharmacologically, interferon-α and JAK inhibitors have shown histological improvements in subsets of patients [152,153]. When the injurious triggers resolve, MET may restore epithelial features; in contrast, persistent inflammatory and mechanical cues tend to stabilize mesenchymal programs and sustain fibrosis [17,105,154].

Consistent with this model, lineage-tracing and single-cell studies in kidney, lung, and liver report little direct epithelial-to-myofibroblast conversion in vivo; instead, pEMT acts mainly through paracrine activation of resident fibroblasts and pericytes to amplify matrix deposition [155,156].

By contrast, in cancer, the process is termed type 3 EMT. Here, genetically and epigenetically altered epithelial tumor cells exploit EMT programs to acquire invasive, metastatic, and therapy-resistant traits [157,158,159]. Unlike the local, paracrine profibrotic role of type 2 EMT in fibrosis, type 3 EMT is often transient or partial (hybrid E/M states), favoring collective migration and allowing cells to revert to an epithelial phenotype through MET at distant metastatic sites [73,157,160]. This plasticity underlies carcinoma dissemination, colonization, and relapse.

Thus, although both type 2 and type 3 EMT share upstream regulators (e.g., TGF-β, WNT, NOTCH, HIPPO), their context-specific outcomes diverge: fibrotic remodeling in chronic organ injury versus invasion and metastasis in cancer [25,147,161].

Recognizing these differences is essential for interpreting lineage-tracing data in fibrosis and for designing therapeutic strategies that target EMT in both oncology and chronic disease.

### 5.3. Functional Role of EMT in Cancer

In cancer, especially epithelial-derived tumors, type 3 EMT is typically partial and transient, enabling invasion, metastatic dissemination, therapy resistance, and phenotypic heterogeneity [67,73,86]. Tumor cells in pEMT frequently occupy hybrid E/M states, retaining junctional and epithelial traits while acquiring motility and stress-tolerance characteristics; this plasticity supports migration and, upon arrival at distant sites, colonization often via MET [11,64,73,147].

EMT programs are frequently partial or transient, conferring collective invasion and enabling MET during metastatic colonization; reversibility is therefore greater during dissemination and micrometastasis than in advanced, therapy-conditioned disease [64,160,162]. Recent clinical-facing and mechanistic syntheses converge on this view, arguing for the timing of anti-plasticity strategies to early disease or minimal residual disease [161,162].

The extent and direction of EMT programs vary across tumor regions, reflecting modulation by paracrine signaling, hypoxia, and interactions with CAFs that remodel the matrix and shape immune exclusion [66,87,88,89]. Functionally, EMT confers evolutionary advantages to malignant clones, including immune evasion, chemoresistance, and enhanced metastatic competence, thereby sustaining progression under therapeutic pressure [67,81,86,147]. Finally, EMT programs intersect with stemness circuits, promoting CSC–like properties. Links to genomic instability have been reported in EMT-high or hybrid states, although the directionality and generality of this relationship remain context-dependent, with chromatin remodeling and replication stress proposed as mediators [13,81,82]. The main differences and similarities between EMT in fibrosis and in cancer are summarized in Table 1.

## 6. Potential Therapies and Future Perspectives

A therapeutic focus on epithelial–mesenchymal programs recognizes EMT as a dynamic and partially reversible circuitry that can be intercepted at multiple levels, from ligands and receptors to mechanotransduction and chromatin control. In oncology, pEMT underlies invasion, metastatic seeding, and treatment tolerance; in fibrotic diseases, pEMT and stromal activation co-stabilize scar-forming states. These shared logics argue for multi-node strategies that can affect plasticity, recalibrate the matrix–mechanosensing axis, and extinguish transcriptional memory, while preserving homeostatic repair [23,105,147,163].

Beyond decelerating progression, true reversal of fibrotic characteristics and outcome likely requires dismantling collagen cross-linking and resetting stromal and epithelial memory [164,165]. Clinically, LOXL2 blockade has not reversed established human fibrosis in IPF or NASH (simtuzumab negative trials) [166,167], whereas etiologic therapy in viral hepatitis and THR-β agonism in MASH have yielded histologic improvement [150]. In metabolic liver disease, the THR-β agonist resmetirom achieved ≥1-stage fibrosis improvement versus placebo in a phase 3 trial [168]. On another front, preclinical inhibition of YAP–TEAD and LOX can reverse profibrotic epithelial reprogramming and attenuate fibrosis in lung models, nominating integrin–FAK–YAP/TEAD axes for future reversal-oriented trials [169,170]. In hematologic diseases, interferon-α and JAK inhibitors (such as momelotinib and ruxolitinib) have shown improvements in histological bone marrow fibrosis grade in subsets [152,153]. Taken together, these patterns support a gradient of reversibility that diminishes as collagen cross-linking and mechano-epigenetic memory accumulate.

The TGF-β axis remains a central target across cancer and fibrosis. Small-molecule ALK5 inhibitors such as galunisertib and ligand/receptor-level approaches are being explored clinically, typically as part of combinations to relieve EMT-linked invasion and immunosuppression or to curb stromal activation. However, safety and on-target pleiotropy demand context-specific modulation rather than broad suppression. Recent reviews synthesize this landscape and frame where TGF-β blockade fits in rational regimens [23,25,171]. As a proof of concept for ligand-selective modulation within the TGF-β superfamily, Activin A–inhibitory peptides identified by phage display bound the receptor-interacting domain of Activin A and, in pulmonary models, suppressed EMT in alveolar epithelium and FMT in fibroblasts, while reducing migration in vitro, supporting the idea that selective ligand targeting may halt EMT and FMT without indiscriminate TGF-β inhibition [172]. In parallel, approved antifibrotics for IPF, pirfenidone and nintedanib, slow lung-function decline but rarely reverse established scarring, reinforcing the need to pair antifibrotics with plasticity- and matrix-focused agents [136,173].

In terms of mechanosignaling and matrix interface, matrix stiffening, integrin–FAK–Rho signaling, and nuclear YAP/TAZ activity cooperate to stabilize mesenchymal bias and EMT-linked states in injured epithelia and activated fibroblasts. Targeting this feedback, alone or layered onto TGF-β-directed therapy, has emerged as a priority across organs, with recent syntheses outlining how YAP/TAZ and upstream mechanosensors shape fibrotic persistence and EMT plasticity [17,169].

On the epigenetic front, persistence of EMT-like cell states reflects chromatin remodeling and ncRNA programs that maintain stemness, drug tolerance, and profibrotic memory. In cancer, EZH2 and related chromatin writers are being pursued clinically to blunt CSC-linked plasticity when combined with chemo-targeted or immune therapy. In fibrosis, BET/BRD4 inhibition has shown broad preclinical antifibrotic activity and is being refined through delivery innovations, although no BET inhibitor has yet been approved for fibrotic indications [108,174,175].

Another therapeutic approach is based on regeneration-aligned strategies. Regenerative approaches seek to reprogram the microenvironment without exacerbating pathologic EMT. Mesenchymal stromal cell–derived extracellular vesicles are gaining traction as cell-free platforms that modulate inflammation, dampen myofibroblast activation, and support epithelial repair. A first-in-human nebulized hUCMSC-EV study in pulmonary fibrosis reported acceptable safety and signals of functional improvement, complementing preclinical data across organs [121,176].

Recent reviews outline the manufacturing, delivery, and quality control considerations for clinical translation of MSC-EVs in lung, liver, and kidney disease [177,178].

Durable benefits will likely depend less on single-pathway blockade and more on biomarker-guided combinations, timed to windows of heightened lesion plasticity: attenuating EMT or pEMT programs, normalizing matrix mechanics and integrin signaling, addressing fibroblast subset heterogeneity, and disabling epigenetic memory, all layered onto standard backbones, for example, antifibrotics in IPF and systemic therapy and immunotherapy in cancer. The shared goal is to modulate plasticity enough to enable directed regeneration and improved performance of foundational therapies, without compromising physiological repair.

## 7. Conclusions

EMT and cellular plasticity emerge as unifying principles across cancer and fibrotic diseases, underpinning key pathological features such as invasion, metastasis, fibroblast activation, therapy resistance, and maladaptive tissue remodeling. Although these processes operate through shared molecular regulators, including TGF-β, WNT, NOTCH, HIPPO, and core EMT transcription factors, their outcomes diverge according to context: uncontrolled proliferation and dissemination in tumors versus chronic scarring and organ dysfunction in fibrosis. Importantly, the recognition of intermediate and reversible EMT states highlights the dynamic and heterogeneous nature of these transitions, offering both challenges and opportunities for therapeutic intervention. Advances in single-cell and spatial omics, combined with lineage-tracing technologies, are now providing unprecedented resolution to map these trajectories in vivo, enabling the identification of context-specific vulnerabilities. Looking ahead, the integration of anti-EMT agents, modulators of epigenetic plasticity, and regenerative strategies may allow not only the containment of cancer progression but also the reversal of fibrotic remodeling.

## Figures and Tables

**Table 1:** Summary Table.

Aspect	Fibrosis	Cancer
**Type of EMT**	Type 2	Type 3
**Stimulus**	Chronic injury, persistent inflammation	Oncogenic signaling, tumor microenvironment
**Phenotype**	Myofibroblastic, ECM-secreting	Invasive, migratory, chemoresistant
**State Stability**	Relatively stable	Highly plastic and reversible
**Functional Role**	Pathological tissue remodeling and wound healing	Tumor progression, metastasis, and therapeutic escape
**Reversibility (MET)**	Limited	Frequent (especially during metastatic colonization)
**Associated Stem Cells**	Poorly characterized	EMT promotes CSC-like features
